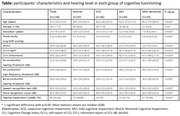# Audiometric hearing level in individuals with SCD, MCI and mild dementia

**DOI:** 10.1002/alz.092317

**Published:** 2025-01-09

**Authors:** Chatchawan Rattanabannakit, Poramad Sirikuntharamas, Triphoom Suwanwech, Sarun Prakairungthong, Witsarut Nanthasi, Vorapun Senanarong

**Affiliations:** ^1^ Faculty of Medicine Siriraj Hospital, Mahidol University, Bangkok Thailand

## Abstract

**Background:**

Hearing impairment is identified as a risk factor for dementia. This study aimed to investigate audiometric hearing level in individuals across the clinical spectrum of cognitive impairment, from normal cognition (NC) to mild dementia, encompassing subjective cognitive decline (SCD) and mild cognitive impairment (MCI).

**Method:**

135 participants were included in this study. There were 33 NC participants, 36 participants with SCD, 34 participants with MCI, and 33 participants with mild dementia. Demographic data was collected, along with the Montreal Cognitive Assessment (MoCA), and the Cognitive Change Index to capture subjective cognitive complaints from patients themselves (CCI‐S) and their informants (CCI‐I). All participants underwent tonal audiometry to measure the best air‐conduction and bone‐conduction hearing threshold, speech recognition test (SRT) and word recognition score (WRS). Association of audiometric data and cognitive function and their differences between diagnostic groups were analyzed.

**Result:**

The median age of all participants was 61.5 (IQR = 19) years, 77.2% were female, median years of education was 14.5 (IQR = 7) years. There were significant differences in age, education, and visual acuity among groups (all p<0.001) (Table). Air‐conduction and bone‐conduction hearing thresholds demonstrated significant differences between groups (all p<0.001), which the worst performance observed in the mild dementia group (p<0.001), even after controlling for age and education (p = 0.013). 63.2% of all participants were defined as having hearing impairment according to WHO criteria, peaking at 93.94% in mild dementia patients. Individuals with moderate hearing impairment demonstrated lower MoCA score and higher CCI‐I scores, compared to those with mild or no hearing impairment. The higher air‐conduction, bone‐conduction hearing threshold, SRT, WRS were significantly correlated with the lower score of MoCA (r = ‐0.47 to ‐0.36, all p<0.001) and a higher score of CCI‐I (r = 0.31 to 0.39, all p<0.001), but not the CCI‐S. However, these associations became insignificant after controlling for age (all p>0.05).

**Conclusion:**

Our study indicates a link between diminishing audiometric hearing levels and a higher degree of objective cognitive decline, along with an increased level of informant perception regarding individuals' cognitive deficits. Not related to age or education, individuals with mild dementia display significantly worse hearing than those without.